# Performance of Commercially Available Serological Screening Tests for Human T-Cell Lymphotropic Virus Infection in Brazil

**DOI:** 10.1128/JCM.00961-18

**Published:** 2018-11-27

**Authors:** Vanessa da Silva Brito, Fred Luciano Neves Santos, Noilson Lazaro Sousa Gonçalves, Thessika Hialla Almeida Araujo, Davi Silva Vale Nascimento, Felicidade Mota Pereira, Ney Cristian Amaral Boa-Sorte, Maria Fernanda Rios Grassi, Adele Caterino-de-Araujo, Bernardo Galvão-Castro

**Affiliations:** a Integrated and Multidisciplinary HTLV Center, Bahiana School of Medicine and Public Health (EBMSP), Salvador, Bahia, Brazil; b Advanced Laboratory of Public Health, Gonçalo Moniz Institute (IGM), FIOCRUZ-BA, Salvador, Bahia, Brazil; c Gonçalo Moniz Public Health Central Laboratory (LACEN-BA), Bahia State Department of Health, Bahia, Brazil; d Adolfo Lutz Institute, São Paulo, São Paulo, Brazil; Memorial Sloan Kettering Cancer Center

**Keywords:** diagnostic reagent kits, HTLV, screening tests

## Abstract

Serological screening for human T-cell lymphotropic virus type 1 (HTLV-1) is usually performed using enzyme-linked immunosorbent assay (ELISA), particle agglutination, or chemiluminescence assay kits. Due to an antigen matrix improvement entailing the use of new HTLV antigens and changes in the format of HTLV screening tests, as well as newly introduced chemiluminescence assays (CLIAs), a systematic evaluation of the accuracy of currently available commercial tests is warranted. We aimed to assess the performance of commercially available screening tests for HTLV infection diagnosis. A diagnostic accuracy study was conducted on a panel of 397 plasma samples: 200 HTLV-negative plasma samples, 170 HTLV-positive plasma samples, and 27 plasma samples indeterminate by Western blotting (WB). WB-indeterminate samples (i.e., those yielding no specific bands for HTLV-1 and/or HTLV-2) were assessed by PCR, and the results were used to compare agreement among the commercially available ELISA screening tests. For performance analysis, WB-indeterminate samples were excluded, resulting in a final study panel of 370 samples. Three ELISA kits (Murex HTLV-1/2 [Murex], anti-HTLV-1/2 SYM Solution [SYM Solution], and Gold ELISA HTLV-1/2 [Gold ELISA]) and one CLIA kit (Architect rHTLV-1/2) were evaluated. All screening tests demonstrated 100% sensitivity. Concerning the HTLV-negative samples, the SYM Solution and Gold ELISA kits had specificity values of >99.5%, while the Architect rHTLV-1/2 test presented 98.1% specificity, followed by Murex, which had a specificity of 92.0%. Regarding the 27 samples with WB-indeterminate results, after PCR confirmation, all ELISA kits showed 100% sensitivity but low specificity. Accuracy findings were corroborated by the use of Cohen's kappa value, which evidenced slight and fair agreement between PCR analysis and ELISAs for HTLV infection diagnosis. Based on the data, we believe that all evaluated tests can be safely used for HTLV infection screening.

## Abstract (precis)

Serological screening for human T-cell lymphotropic virus type 1 (HTLV-1) is usually performed using enzyme-linked immunosorbent assay (ELISA), particle agglutination, or chemiluminescence assay kits. Due to an antigen matrix improvement entailing the use of new HTLV antigens and changes in the format of HTLV screening tests, as well as newly introduced chemiluminescence assays (CLIAs), a systematic evaluation of the accuracy of currently available commercial tests is warranted.

## INTRODUCTION

Human T-cell lymphotropic virus type 1 (HTLV-1) and type 2 (HTLV-2) were identified in 1980 and 1982, respectively (1, 2). Subsequently, HTLV-3 and HTLV-4 were discovered in 2005 (3, 4). It has been estimated that at least 10 million people harbor HTLV-1 worldwide (5). Large foci of infection with this virus exist in Japan, Africa, the Caribbean Islands, Melanesia, Australia, the Mashhad area of northeastern Iran, and South America (5–7). HTLV-1 is associated with or causes a broad range of inflammatory conditions and a severe proliferative disease (5, 8–14).

HTLV-2 infection is endemic in native Amerindian populations in both North and South America, certain tribes of Pygmies in Africa, and intravenous drug users (IDUs) in urban areas around the world (15, 16). In contrast to HTLV-1, this type is rarely associated with neurological or lymphoproliferative disorders (17). HTLV-3 and HTLV-4 are restricted to western Africa and have not yet been associated with any diseases (3, 4).

Brazil, a country of 200 million inhabitants, has a population of 800,000 who potentially harbor HTLV-1, representing one of the largest areas of endemicity for the virus and its associated diseases anywhere in the world (5). The virus is disseminated throughout the country, with higher rates of infection being found in the northeast and northern regions than in the south and southeast (18, 19). HTLV-2 is present mainly in the north, among indigenous populations, and in IDUs in urban centers (17).

Achieving an accurate diagnosis of HTLV infection is a complex task. Serological screening for HTLV-1 is usually performed using enzyme-linked immunosorbent assay (ELISA), particle agglutination testing, or chemiluminescence assay (CLIA) kits. The Brazilian Ministry of Health recommends the use of ELISA or particle agglutination tests as a screening protocol. Western blotting (WB), or immunoblotting, is used for confirmation, and PCR is employed in the case of inconclusive confirmatory test results (20). Among the screening options, ELISA is used most extensively due to an elevated level of automation, simplicity, and low cost. ELISA performance depends on the antigen composition and assay format (21–24). Tests providing low accuracy present a public health problem, as false-positive results can have a negative impact not only in economic terms, due to the need for confirmation by WB, but also on individuals' quality of life.

In light of this scenario, we endeavored to conduct a systematic evaluation of the performance of commercial screening test kits for HTLV infection diagnosis. Statistical tools were used to obtain a robust assessment of the performance of each serological test by determining the following diagnostic test parameters: sensitivity (the probability of a test being positive in the presence of infection) and specificity (the probability of a test being negative in the absence of infection).

## MATERIALS AND METHODS

### Ethical considerations.

The present research protocol was approved by the Institutional Research Board (IRB) of the Bahiana School of Medicine and Public Health (EBMSP) in Salvador, Bahia, Brazil (protocol no. 464.286). All procedures were performed in accordance with the principles established in the Declaration of Helsinki and its subsequent revisions.

### Sample selection.

The present diagnostic accuracy study was carried out between February 2015 and December 2017 using anonymous plasma samples obtained from the biorepository of the Integrated and Multidisciplinary HTLV Center (CHTLV) at EBMSP. CHTLV is an outpatient clinic, open to the public, that provides interdisciplinary care and services, including general medical treatment, laboratory diagnosis, psychological counseling, and physical therapy. All included plasma samples had previously been screened for antibodies against HTLV-1/2 using an enzyme-linked immunosorbent assay (Ortho HTLV-1/HTLV-2 Ab-Capture ELISA systems; Ortho-Clinical Diagnostic, Raritan, NJ, USA), and reactive samples were retested by Western blotting (HTLV Blot, version 2.4; Genelabs Diagnostics, Singapore). Test results were interpreted according to the stringent criteria indicated by the manufacturer and in accordance with the guidelines established by the Brazilian Ministry of Health (20).

**FIG 1:**
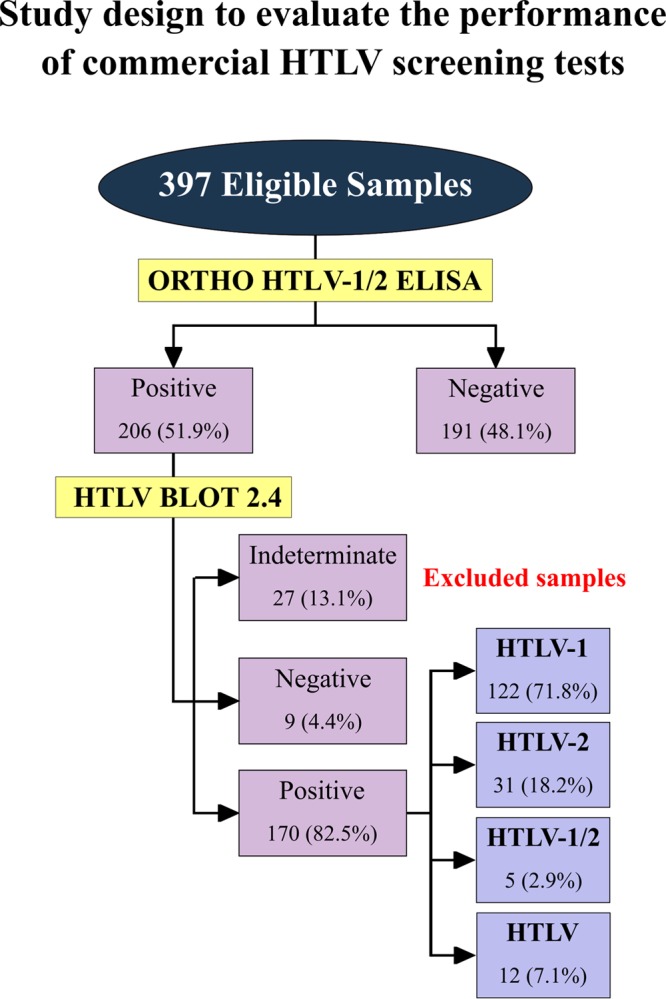
Flowchart depicting the study design, in accordance with Standards for Reporting of Diagnostic Accuracy Studies (STARD) guidelines.

The panel consisted of 397 samples: 200 HTLV-negative, 170 HTLV-positive (122 HTLV-1-positive, 31 HTLV-2-positive, 5 HTLV-1 plus HTLV-2-positive, and 12 HTLV-positive), and 27 WB-indeterminate plasma samples. Briefly, HTLV-negative samples were defined as those lacking reactivity to HTLV-specific proteins; HTLV-1-positive samples were defined as those reactive to Gag (p19 with or without p24) and two Env proteins (GD21 and rgp46-I); HTLV-2-positive samples were those reactive to Gag (p24 with or without p19) and two Env proteins (GD21 and rgp46-II); HTLV-seropositive samples were those reactive to Gag (p19 and p24) and Env (GD21); and samples were considered indeterminate when no HTLV-specific bands were detected; i.e., the criteria for HTLV-1, HTLV-2, or HTLV were not satisfied. Indeterminate samples were assessed by PCR analysis, and the results obtained were used to compare the agreement with ELISA screening test results. For performance analysis, the WB-indeterminate samples were excluded, forming a final study panel of 370 samples (Fig. 1).

Alternatively, 217 plasma samples (112 positive, 105 negative) were also assessed by a chemiluminescence assay (CLIA; Architect rHTLV-1/2; Abbott Diagnostics Division, Wiesbaden, Germany).

### Immunoassays.

Three HTLV-1/2-specific enzyme-linked immunosorbent assay kits, all commercially available in Brazil, were employed in this study: Murex HTLV-1/2 (Murex; DiaSorin S.p.A., Dartford, UK), anti-HTLV-1/2 SYM Solution (SYM Solution; Symbiosis Diagnostica LTDA, Leme, Brazil), and Gold ELISA HTLV-1/2 (Gold ELISA; Rem Indústria e Comércio LTDA, São Paulo, Brazil). Cutoff values, as well as gray zones, were calculated for each test as follows: by adding 0.2 to the mean for the negative-control replicates for Murex HTLV-1/2, by adding 0.18 to the mean for the negative-control replicates for anti-HTLV-1/2 SYM Solution, and by adding 0.25 to the mean for the negative-control replicate for Gold ELISA HTLV-1/2. For data normalization, all results were expressed by plotting values in an indexed format, calculated as the ratio between a given sample's optical density (OD) and the cutoff OD values for each assay. Under this index, referred to as a reactivity index (RI), all results with an RI of <1.00 were considered negative. When a sample's RI value was 1.0% ± 10%, the result was considered indeterminate (i.e., in the gray zone), and the results for these samples were deemed inconclusive.

### HTLV-1/2 molecular detection.

Peripheral blood mononuclear cells (PBMC) from 27 patients with WB-indeterminate results were obtained from EDTA blood samples under density gradient centrifugation; DNA was extracted using a spin column kit (Qiagen, Hilden, Germany). DNA samples were submitted to nested PCR using HTLV-1 long terminal repeat (LTR) 5′ region-specific primers as described previously (25), outer primers BSQF6 and BSDR3, and inner primers BSQF2 and BSDR4 to amplify a 672-bp fragment in the HTLV-2 LTR region (26). All amplified products were submitted to electrophoresis on a 1% agarose gel with Syber Safe DNA (Invitrogen).

### Statistical analysis.

Data were encoded and analyzed using scatterplot computer graphic software (Prism, version 7; GraphPad, San Diego, CA). Descriptive statistics are presented as the geometric means ± standard deviations. To test data normality, the Shapiro-Wilk test, followed by Student's *t* test, was used. When the assumed homogeneity was not confirmed, Wilcoxon's signed-rank test was used. All analyses were two-tailed, and *P* values under 5% were considered significant (*P* < 0.05). Enzyme-linked immunosorbent assay performance was computed using a dichotomous approach, and the performance characteristics of the various assays were compared in terms of sensitivity, specificity, accuracy, likelihood ratio (LR), and diagnostic odds ratio (DOR). Additionally, receiver operating characteristic (ROC) curves were constructed, and the areas under these curves were used as a global measure of test performance. Confidence intervals (CI) were employed at a confidence level of 95%. The strength of agreement between the results of the screening commercial tests and the PCR results was assessed by the use of Cohen's kappa coefficient (κ) (27), which accounts for agreement occurring only by chance beyond simple percent agreement calculations. κ values are interpreted as poor (κ ≤ 0), slight (0 < κ ≤ 0.20), fair (0.21 < κ ≤ 0.40), moderate (0.41 < κ ≤ 0.60), substantial (0.61 < κ ≤ 0.80), and almost perfect (0.81 < κ ≤ 1.0) agreement. A flowchart (Fig. 1) has been provided depicting study design in accordance with the Standards for Reporting of Diagnostic Accuracy Studies (STARD) guidelines (28).

## RESULTS

### Assay performance.

**FIG 2:**
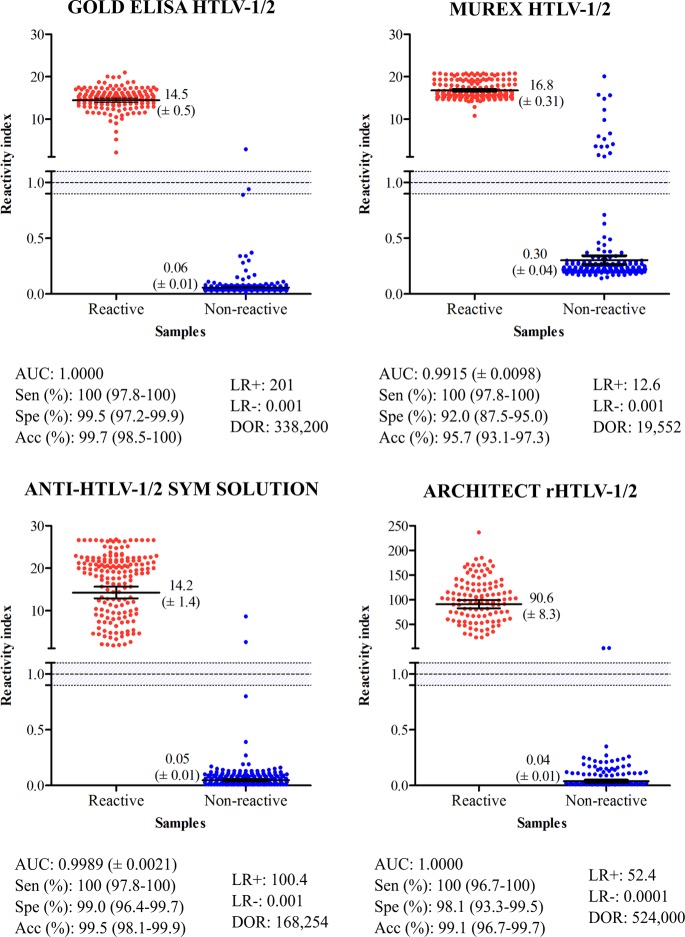
Reactivity index of screening assays obtained with positive (red dots) and negative (blue dots) plasma samples by WB analysis for HTLV-1/2. The cutoff value was an IR of 1.0, and the area delimited by lines represents the indeterminate zone (RI ± 10%). The numbers shown for each group represent geometric means (±95% CI). AUC, area under curve; Sen, sensitivity; Spe, specificity; Acc, accuracy; LR, likelihood ratio; DOR, diagnostic odds ratio.

Using plasma from 170 HTLV-positive individuals, the performance characteristics of the ELISAs and CLIA were assessed, as shown in Fig. 2. The area-under-the-curve (AUC) values were >99%, demonstrating excellent overall diagnostic accuracy for all kits tested. RI values for HTLV-1/2-positive samples were variable, ranging from 14.2 for SYM Solution and 14.5 for the Gold ELISA to 16.8 for Murex. In addition, Architect rHTLV-1/2 yielded the highest RI value (>90).

As all test kits demonstrated 100% sensitivity, no statistically significant differences were detected. Regarding the HTLV-1/2-negative samples, SYM Solution and Gold ELISA presented specificity values of >99%. Architect rHTLV-1/2 showed a specificity of 98.1%, followed by Murex, which showed a specificity of 92.0%. The differences in specificity and RI between the SYM Solution and Gold ELISA kits were not statistically significant. With respect to the HTLV-negative samples, the maximum RI value was obtained using Murex (RI = 0.30) (Fig. 2). When RI values of 1.0 ± 0.10 were considered inconclusive, i.e., the RI values fell in the gray zone, we verified that samples truly positive for HTLV-1/2 were conclusively detected by the Gold ELISA, Murex, SYM Solution, and Architect rHTLV-1/2 tests. As regards the HTLV-negative samples, one fell in the gray zone using the Gold ELISA. With respect to diagnostic accuracy, the Gold ELISA, SYM Solution, and Architect rHTLV-1/2 tests demonstrated the highest accuracy (>99.1%), while Murex presented the lowest accuracy result (95.6%). DOR scores, based on likelihood ratios, were 524,000 for Architect rHTLV-1/2, 338,200 for Gold ELISA, 168,254 for SYM Solution, and 19,552 for Murex HTLV-1/2. Among the ELISA kits evaluated, Gold ELISA offered the best performance, as evidenced by ROC analysis and, notably, the exceptionally high diagnostic odds ratio produced by this test (Fig. 2). No significant differences in RI signals were observed with regard to the different types of seroreactivity (HTLV-1 versus HTLV-2 versus HTLV-1/2 and HTLV).

### Assay agreement.

**FIG 3:**
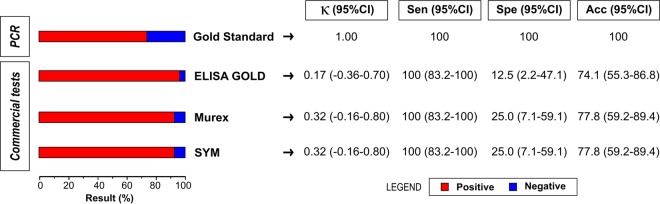
Analysis of WB-indeterminate samples using PCR as a gold standard. Acc, accuracy; CI, confidence interval; κ, Cohen's kappa coefficient; Sen, sensitivity; Spe, specificity.

Analysis of the diagnostic accuracy of the three commercial ELISAs with respect to the 27 WB-indeterminate samples, considering PCR amplification as the gold standard for HTLV infection diagnosis, revealed that 8 samples were negative (29.6%) and 19 were positive (70.4%) for HTLV-1 (Fig. 3), with all ELISAs yielding 100% sensitivity. Conversely, all three assays presented specificity inferior to 25%, with Gold ELISA offering just 12.5% specificity. Despite this very low accuracy, both the Murex and SYM Solution kits offered higher accuracy than Gold ELISA. Slight and fair agreement (Cohen's kappa value < 0.40) between the results of PCR analysis and the ELISA screening tests for the diagnosis of HTLV infection was detected. Table 1 details the 27 HTLV-indeterminate profiles that allowed for the identification of distinct patterns. No HGIP (29) or N (30) patterns were observed.

**TABLE 1:** Indeterminate HTLV patterns in samples from Brazil

WB pattern (reference)	No. (%) of samples	No. of samples positive by the following assay/total no. of samples tested:		
		Gold^*b*^	Murex^*c*^	SYM^*d*^
gd21 alone	7 (25.9)	7/7	6/7	6/7
gd21 + p19	7 (25.9)	7/7	7/7	7/7
gd21 + a synthetic peptide (46I or 46II)	5 (18.5)	4/5	4/5	4/5
Others^*a*^	8 (29.7)	8/8	8/8	8/8
HGIP^*e*^ (29)	0			
N^*f*^ (30)	0			
Total	27 (100)	26/27	25/27	25/27

a One band each for gd21, p19, and p28; gd21, p19, p26, p28, and p32; gd21, p19, p28, and p36; gd21, p19, p26, p28, and p36; gd21, p19, p26, p28, p32, and p36; p19, p21, p26, p28, p32, p36, MTA-1, and pr53; p19, p26, and p28; and synthetic peptide 46II alone.

b Gold ELISA HTLV-1/2 (Rem Indústria e Comércio LTDA, São Paulo, Brazil).

c Murex HTLV-1/2 (DiaSorin S.p.A., Dartford, UK).

d Anti-HTLV-1/2 SYM Solution (Symbiosis Diagnostica LTDA, Leme, Brazil).

e HGIP, HTLV-I Gag indeterminate Western blot pattern.

f N, new pattern.

## DISCUSSION

The present study found a high diagnostic value for each of the four different commercially available HTLV screening tests evaluated for the detection of anti-HTLV antibodies in Brazil. In fact, AUC values greater than 99% demonstrate convincing evidence of the optimal discriminative power of these kits regarding HTLV-positive and HTLV-negative samples. Both Gold ELISA and Architect rHTLV-1/2 presented AUC values of 100%. Furthermore, the Murex, SYM Solution, and Architect rHTLV-1/2 assays did not show inconclusive results (gray zone) in HTLV antibody screening procedures. The Gold ELISA yielded a low number of inconclusive results, as only 1 out of 170 HTLV-positive samples tested using this kit produced an RI value that fell in the gray zone.

All tests displayed 100% sensitivity in detecting HTLV-positive samples. RI values were higher than 14 for the ELISAs and above 90 for the Architect rHTLV-1/2 kit, which corroborates previous reports (31). Regarding the ELISAs, the highest RI value was achieved by Murex, with statistically significant differences from the results of the Gold ELISA and SYM Solution assays being seen.

Due to the high number of samples (4.3%) misdiagnosed by the Murex test, its accuracy was significantly lower than that of the other kits. Gold ELISA, SYM Solution, and Architect rHTLV-1/2 were all found to be 99% accurate, suggesting that these kits can be safely employed for HTLV infection screening. Although the Murex test was less accurate, it nonetheless returned values above 95%, indicating suitability for the diagnosis of HTLV infection; however, the proportion of samples requiring WB confirmation was greater, which increases the cost of performing a diagnosis. In fact, 8% of the HTLV-negative samples assayed with Murex yielded false-positive results, with a specificity of 92%. It is interesting to note that this test's performance has improved over time, as studies performed in 2007 and 2009 described its sensitivity and specificity to be 98.2% and 42.6%, respectively (32, 33). Another study conducted in Argentina showed that Murex was 97.2% sensitive and 99.7% specific (34). More recently, other studies have reported high values of specificity; these studies included those evaluating HIV/HTLV-coinfected individuals (99.0%) (31) and blood donors (97.2%) (34). With respect to HTLV-negative samples, the Murex test returned the highest RI value. The observed differences in RI values could arise from variability in antigenic composition. While all tests correctly detected positive samples, it is possible that the antigenic matrix employed in the solid phase of the Murex kit recognized no specific anti-HTLV antibodies, which led to false-positive results or cross-reactions.

Of note, the sensitivity, specificity, and accuracy values associated with diagnostic tests are unsatisfactory in terms of influencing clinical decisions (35). A diagnostic test can be considered valid only if the results produced modify the probability of disease occurrence. Likelihood ratio (LR) measurements can be helpful in describing a test's discriminatory power and determining the probability that a particular result will occur among infected individuals, as opposed to the probability that the same result will be obtained among healthy individuals (36). In our study, Gold ELISA had a positive LR of 201, indicating that an HTLV-infected person is approximately 201 times more likely to be diagnosed with this infection if evaluated with this kit. The lowest positive LR value was observed with the Murex test (12.6), indicating a low probability that infection in an HTLV-infected person will be accurately diagnosed. Conversely, a study performed in 2008 found a positive LR of 326.5 for Murex (34). HTLV-negative samples returned LR values of less than 0.001 for all of the evaluated tests. There is a consensus that positive LR values above 10 and negative LR values below 0.1 contribute substantially to the diagnosis (36). DOR, calculated as the ratio between positive and negative LR values, is considered a global performance parameter that summarizes the diagnostic test accuracy. DOR values describe the probability of receiving a positive result for a person with infection, as opposed to someone who is uninfected (35). The DOR for Architect rHTLV-1/2 (524,000) was the highest among the screening tests evaluated, followed by Gold ELISA (338,200), SYM Solution (168,254), and Murex (19,552). These findings suggest that Architect rHTLV-1/2 and Gold ELISA offer performance superior to that of SYM Solution and Murex. LR and DOR determinations are relevant, and these are stable tools, since these parameters remain independent of the prevalence of disease (37). The HTLV-1- and HTLV-2-seroindeterminate WB patterns observed herein were similar to those reported by other studies. However, no HGIP or N patterns were identified.

It is important to note that, concerning the Architect rHTLV-1/2 test, our findings are in agreement with those reported by other studies. In fact, identical values of sensitivity (100%) and specificity (>99%) have been described for samples from both blood donors and hospitalized patients (38). Similar results were demonstrated by Malm et al. (39) (sensitivity, 100%; specificity, 99.8%), as well as by Qiu et al. (40) (sensitivity, 100%; specificity, 99.98%) in general populations in the United States, Japan, and Nicaragua. Although the present study was unable to assess other screening tests, the literature indicates the high performance of both the Elecsys HTLV-I/II and Abbott Prism HTLV-I/HTLV-II kits (sensitivity, 100%; specificity, >99%) both in samples from blood donors and in other samples obtained from a routine diagnostic service (41). The DiaSorin Liaison XL recHTLV-I/II kit was also evaluated elsewhere, with high sensitivity and specificity values being reported, similar to the findings for the Architect rHTLV-1/2 test (42–44).

The results presented herein indicate that all evaluated kits can safely be used for HTLV infection screening. However, it is important to note that the high sensitivity offered by these kits may lead to false-positive results, which could increase the cost of testing as a result of WB confirmation requirements. From the perspective of large diagnostic centers and blood banks, proper screening method selection can substantially reduce the costs associated with confirmatory testing. In an effort to reduce costs and ensure a correct diagnosis, a new diagnostic protocol for HTLV infection diagnosis was proposed by Costa et al. (45), who suggested the use of two ELISAs for screening purposes, followed by real-time PCR. In this case, confirmation by WB would be indicated only in cases of negative PCR results. Herein, when the 27 WB-indeterminate samples were analyzed by PCR, the results for all HTLV-1-positive samples demonstrated agreement with the results from each of the three ELISAs evaluated. On the other hand, overall agreement was slight or fair due to the high number of false-positive results obtained using ELISA. Moreover, it has been demonstrated that the INNO-LIA HTLV I/II Ab serological confirmatory assay for HTLV yielded results for most of the samples considered indeterminate or positive, but untypeable, in WB assays (31, 46). These data suggest that the costs associated with HTLV infection diagnosis could be lowered by using molecular biology-based methodologies or INNO-LIA HTLV I/II Ab as a confirmatory assay in place of WB. In the context of low-income countries, such as those in Africa and Latin America, we suggest that CLIA represents a suitable screening strategy for blood banks due to the high DOR values found herein. However, in countries lacking the necessary infrastructure, the use of an ELISA offering a high DOR value, e.g., Gold ELISA, seems to be a satisfactory alternative.

Despite the scarcity of studies evaluating the diagnostic performance of screening tests in diagnosing HTLV infection by employing LR, DOR, and AUC as performance parameters, we evaluated three ELISAs and one CLIA for HTLV infection screening. Based on the present findings, we conclude that all of the commercially available 3rd-generation kits employed herein presented sensitivity and specificity values higher than those found in previous studies. Among the ELISAs evaluated, the Gold ELISA HTLV-1/2 kit offered the best performance parameters, while Architect rHTLV-1/2 demonstrated the highest performance of all the assays considered. The high sensitivity values produced by screening tests could lead to high proportions of false-positive results. Thus, we reinforce our previous suggestion and urge the consideration of a new protocol employing molecular biology or line immune assay (INNO-LIA HTLV I/II Ab) techniques as a first choice for confirmatory testing in place of WB.
